# Editorial: Plant specialized metabolisms: physiological responses and molecular mechanisms

**DOI:** 10.3389/fpls.2025.1629842

**Published:** 2025-06-17

**Authors:** Xinru Cui, Weiwei Zhang, Xiaomeng Liu, Feng Xu, Moonhyuk Kwon

**Affiliations:** ^1^ College of Horticulture and Gardening, Yangtze University, Jingzhou, China; ^2^ School of Modern Industry for Selenium Science and Engineering, National R&D Center for Se-rich Agricultural Products Processing Technology, Wuhan Polytechnic University, Wuhan, China; ^3^ Division of Applied Life Science (BK21 Four), Anti-Aging Bio Cell Factory Regional Leading Research Center, Research Institute of Molecular Alchemy, Gyeongsang National University, Jinju, Republic of Korea

**Keywords:** specialized metabolites, biosynthetic mechanisms, regulatory networks, environmental adaptations, transcription factors

Plant specialized metabolites (PSMs), also referred to as “secondary metabolites”, encompass a wide range of compounds such as flavonoids, quinones, terpenoids, alkaloids, and phenolic compounds. These metabolites have evolved to perform specific physiological and ecological roles, significantly contributing to plant adaptation under diverse environmental conditions, stress resistance, development processes, and metabolic regulation. Notably, certain PSMs possess physiological activities and pharmacological effects that hold promise in the prevention and treatment of various human diseases, including cancer, aging-related disorders, and cardiovascular conditions. Additionally, some PSMs have valuable industrial applications, such as natural rubber. The biosynthesis of PSMs exhibits remarkable diversity and complexity, varying across species, organs, tissues, and developmental stages. This biosynthetic complexity is governed by genetic factors and tightly regulated through interactions among transcription factors (TFs), *cis*-regulatory elements, and environmental cues (both biotic and abiotic stimuli).

Elucidating the biosynthetic pathways and gene functions associated with specific metabolites is essential for understanding their regulatory mechanisms. For instance, Long et al. employed comparative metabolomics across wolfberry and six other species (maize, rice, wheat, soybean, tomato, and grape), identifying 16 metabolites specific to wolfberry. By comparing the copy number of key enzymes in metabolite synthesis and degradation, they found that the phenyllactate degradation gene *UGT1* had the lowest copy number among the six species, whereas the riboflavin and phenyllactate synthesis genes *RFK* and *HPPR* had higher copy numbers. This suggests that the copy numbers of *RFK, HPPR*, and *UGT1* may be the main reasons for the specific accumulation of riboflavin and phenyllactate in wolfberry. In *Rubus chingii*, Xu et al. identified 32 RcBBX transcriptional factors. By integrating BBX expression profiles across organs (roots, stems, leaves, flowers, fruits), developmental stages, and abscisic acid (ABA) treatments, they predicted RcBBX26 as a potential activator of anthocyanin biosynthesis and accumulation in red chestnut fruits. Moreover, the expression trends of seven anthocyanin biosynthetic genes (*Rc4CL4, Rc4CL5, Rc4CL6, Rc4CL12, RcUFGT8, RcUFGT9, and RcUFGT11*) were consistent with *RcBBX26* and anthocyanin accumulation during fruit ripening, indicating that *RcBBX26* positively regulates anthocyanin biosynthesis by activating target gene expression. In mung bean, Cho et al. identified six genes encoding four key enzymes (*CCoAOMT1*, *CYP81E1*, *DFR*, *HCT*), which commonly affect the levels of secondary metabolites (catechin, chlorogenic acid, formononetin, genistin). Regulatory network analysis revealed that NAC042 and MYB74 TFs orchestrate the expression of these enzymes, enhancing flavonoid content. These results could improve the nutritional value of mung beans and contribute to developing high-quality mung bean varieties.

Deciphering the biosynthetic mechanisms of various PSMs and increasing the production of valuable metabolites through gene editing are the key objectives in this field. Functional characterization plays a crucial role in unraveling complex molecular mechanisms. For example, Ma et al. utilized yeast two-hybrid and two-luciferase assays to show that CaMYBA, CaMYC, and CaTTG1 form an MYB-bHLH-WD40(MBW) complex, which directly bind promoters of anthocyanin synthesis structural genes such as *CaANS* to promote transcription and anthocyanin accumulation in pepper leaves. Covarrubias et al. found that overexpression of *SlLIP1* in tomato enhances fruit-specific *SlLIP1* transcripts, accompanied by increased bound and unbound linoleic acid (LA) content in fruit. Targeted metabolomics analysis of polar metabolites using LC-MS/MS showed that the LA content increase was associated with modifications at the level of transcripts of various genes involved in LA biosynthesis. Martinez-Sanchez et al. used RNA-seq and qPCR to analyze anthocyanin profiles and the expression of genes encoding anthocyanin biosynthetic enzymes revealed that SlATV represses anthocyanin biosynthesis by inhibiting key gene expression. Transient and stable transformation showed that pepino R2R3 MYB113 (AN1-like) is a key transcriptional activator for pepino anthocyanin accumulation. The study by Wolters et al. elucidated the interaction of dandelion TkSRPP with TkUGT80B1 providing a new connection between TkSRPPs and triterpenoid saponin synthesis in *T. koksaghyz* latex. This result will help to further elucidate the network of proteins linking TkSRPPs, stress responses, and NR biosynthesis within the cellular complexity of latex.

Plant adaptation to environmental changes is closely associated with the dynamic changes in the profiles of PSMs. Therefore, exposing plants to specific induced conditions is an effective way to identify changes in the corresponding metabolites and their regulatory genes. In tobacco, Wei et al. demonstrated the transformation of tobacco metabolites through different roasting processes. The analysis revealed a series of differentially expressed metabolites (DEMs) among fresh leaf, normal curing (NC), excessive curing (EC), and insufficient curing (IC) leaves at the end of 42°C, 54°C, and 68°C, respectively, suggesting that the roasting process regulates the transformation of tobacco metabolites and has a significant effect on the formation of tobacco quality. In addition, LC-MS/MS identified 845 metabolites, with flavonoids as the most abundant class. Petrovic et al. found that the specialized metabolism of *Nepeta nuda* L. leaves is more reprogrammable in response to differential growth conditions than that in inflorescences. Guo et al. combined proteomic and metabolomic analysis of the changes in tobacco leaves under under both topping and non-topping conditions. They found that the expression of proteins such as chalcone synthase (CHS), chalcone isomerase (CHI), naringenin 3-dioxygenase (F3H), and flavonoid 3’-monooxygenase (F3’H) was upregulated, and metabolites like pinocembrin, kaempferol, trifolin, rutin, and quercetin also increased thus enhancing the biosynthetic pathways of “flavonoid” and “flavone and flavonol”.

Evolutionary divergence also shapes metabolite biosynthesis. Farzana et al. revealed and characterized a new ƔTMT-like enzyme, perivine Nb-methyltransferase (TePeNMT) from the plant *Tabernaemontana elegans*, distinguishing it from other ƔTMTs and ƔTMT-like NMTs. Their findings suggest that parallel evolution of ancestral gTMTs may be responsible for the occurrence of perivine N-methylation in *T. elegans* and *Catharanthus. roseus*.

Finally, this Research Topic includes a review by Wang et al. on different factors affecting the synthesis and accumulation of secondary metabolites in *Ficus carica*, including varieties, tissue type, environmental factors (e.g., light), stresses (e.g., high temperature, low temperature, drought, nutrient deficiencies, salinity), hormonal treatments, and developmental factors. Furthermore, they discussed the role of structural genes and TFs in the biosynthesis of secondary metabolites, specifically anthocyanins and furanocoumarins. The results of this research have important application prospects for further research and development of new *F. carica* varieties.

